# Predictors of positive and negative parenting behaviours: evidence from the ALSPAC cohort

**DOI:** 10.1186/1471-2431-14-247

**Published:** 2014-10-03

**Authors:** Rachel M Thomson, Clare S Allely, David Purves, Christine Puckering, Alex McConnachie, Paul CD Johnson, Jean Golding, Christopher Gillberg, Philip Wilson

**Affiliations:** Institute of Health and Wellbeing, University of Glasgow, RHSC Yorkhill, Glasgow, G3 8SJ Scotland; School of Health Sciences, University of Salford, Allerton Building, Frederick Road, Salford, M6 6PU England, UK; Robertson Centre for Biostatistics, University of Glasgow, Boyd Orr Building, Glasgow, G12 8QQ Scotland; Centre for Child and Adolescent Health, School of Social and Community Medicine, University of Bristol, Bristol, England, UK; Centre for Rural Health, The Centre for Health Science, University of Aberdeen, Old Perth Road, Inverness, IV2 3JH Scotland

**Keywords:** Parent-infant interactions, Positive parenting, Negative parenting, Mellow parenting system, ALSPAC

## Abstract

**Background:**

This study aimed to establish the predictors of positive and negative parenting behaviours in a United Kingdom population. The majority of previous research has focused on specific risk factors and has used a variety of outcome measures. This study used a single assessment of parenting behaviours and started with a wide range of potential pre- and post-natal variables; such an approach might be used to identify families who might benefit from parenting interventions.

**Methods:**

Using a case-control subsample of 160 subjects from the Avon Longitudinal Study of Parents and Children (ALSPAC), regression analysis was undertaken to model parenting behaviours at 12 months as measured by the Mellow Parenting Observational System.

**Results:**

Positive parenting increased with maternal age at delivery, levels of education and with prenatal anxiety. More negative interactions were observed among younger mothers, mothers with male infants, with prenatal non-smokers and among mothers who perceived they had a poor support structure.

**Conclusions:**

This study indicates two factors which may be important in identifying families most at risk of negative parenting: younger maternal age at delivery and lack of social support during pregnancy. Such factors could be taken into account when planning provision of services such as parenting interventions. We also established that male children were significantly more likely to be negatively parented, a novel finding which may suggest an area for future research. However the findings have to be accepted cautiously and have to be replicated, as the measures used do not have established psychometric validity and reliability data.

## Background

Parenting encompasses a complex and multi-dimensional set of behaviours influenced by multiple interacting, intra- and inter-personal factors and environments [1, 2], and it is well established that the parenting practices to which children are exposed can impact on their development, future health and social functioning. Early positive parenting is associated with reduced risk for development of conduct disorder [3] and childhood depression [4], and predicts increased empathy and pro-social behaviour [5]. Conversely, negative parenting is associated with adverse developmental trajectories, seen as early as six months and with the performance gap increasing over time [6]; early language and social skill development [7] seem to be at greatest risk. Behaviourally, it is negatively associated with school performance [6], and has been associated with increased antisocial behaviour even after controlling for genetic confounding [8], increased adolescent risky sexual behaviours [9] and substance misuse [10]. In relation to future mental health, negative parenting has been linked with increased risk of developing a broad range of mental health conditions in later life [11].

Early prediction of parenting behaviours may be clinically relevant, as the potential exists to offer intervention before the onset of negative consequences [12]. The long-term efficacy of very early parenting interventions can remain evident many years later [13], but more benefit is achieved with more intensive programmes in initially more distressed families [12]. For maximal cost-effectiveness therefore, identification of an ‘at-risk’ population is desirable [14].

The existing literature within this field is substantial, and there are few consistently applied and reliable outcome measures of parenting behaviours. Combined with the narrow focus of much research on specific risk factors, rather than analysis of many potential variables, comparative evaluation is thus difficult. There is also some divergence within the literature: for example, lower maternal age has most frequently been found to predict negative parenting [2, 15, 16], but Bryanton et al found that a maternal age less than 30 in their population predicted positive parenting [17].

Other predictors of positive parenting include high parental self-efficacy [17, 18] perceived maternal role competence [19] and high maternal learned resourcefulness [19], along with good perceived partner relationship [17], excellent partner support [20] and where the mother had experienced domestic violence but left [21]. Conversely, those mothers who perceived low levels of support in their marriage were more likely to exhibit negative parenting [2]. Increased maternal education has also been associated with positive parenting [20], as has higher socioeconomic status [22], and lower socioeconomic status has also been shown to predict negative parenting [2, 15]. Multiparity has been found to be predictive of both positive parenting [17] and negative parenting [6], again highlighting the need for further study. In relation to the perinatal and postnatal period, a positive perception of the birth experience [17], vaginal birth [20] and maternal perception of infant contentment have also been found to predict positive parenting.

The existing literature on predictors of negative parenting has been somewhat focused on the issues of parental stressors, particularly in relation to parental depression where an association with negative parenting is well established [2, 6, 19, 21, 23–26], but also parental anxiety disorders [2], parental dissociative disorders [21] and maternal substance abuse [27]. Other factors such as increased maternal emotional stress [7], high parental fatigue [28], ineffective coping styles [28] and poor partner choice [29] have also been associated with negative parenting. Interestingly, mothers who were negatively parented themselves seem more likely to exhibit negative parenting towards their offspring [7], as do mothers who were physically abused as children [21]. Socially, negative parenting has also been associated with households where there is limited English spoken by parents, where there are more than three children in the home, where there have been multiple moves [6], and also where there is inadequate social support [28], exposure to violence [24], poor diet or poor sleep quality [28]. Lastly, negative parenting is more likely to be exhibited where parents report high levels of child rebelliousness and disobedience [2], though it is unclear whether one can infer causality from this or if it may be a consequence of existing negative parenting pre-interview.

The current research in this area has been hindered by the narrow focus of research questions and the lack of a uniform outcome measure within the literature. Using a sample from a large UK-based longitudinal birth cohort and a structured assessment of parenting behaviours (the Mellow Parenting Observational System), this study aims to build on existing knowledge of predictors of parenting behaviours to investigate a wide variety of potential predictors within this population, without focus on any particular group of characteristics. We hypothesise that in addition to a range of established factors (for example maternal depression and decreased maternal age), a range of previously unassessed factors may predict parenting behaviours.

## Methods

### Participants

The data were collected as part of the Avon Longitudinal Study of Parents and Children (ALSPAC), an on-going longitudinal birth cohort study which started in the early 1990s. Pregnant women were recruited in the former Avon health authority in south-west England with expected delivery dates between 1st April 1991 and 31st December 1992. The study website contains details of all the data that are available through a fully searchable data dictionary (http://www.bris.ac.uk/alspac/researchers/data-access/data-dictionary/). Enrolment was estimated to be around 80-90%, and the data provide a broad range of biological, environmental, social, psychological and psychosocial exposures and various health and developmental outcomes [30]. Much of this information was collected from participants in the form of questionnaires, and the details of those which are relevant to this particular study are outlined below. Ethical approval for the study was obtained from the ALSPAC Law and Ethics Committee and the Local Research Ethics Committees, and informed consent was obtained from all adult participants prior to their inclusion in the study.

Of the core cohort of 13,988 infants, 10% were randomly selected to be examined in more detail, encompassing 10 examinations between four months and five years [31]. This group, known as ‘Children in Focus’, had an assessment at age 12 months which included the Thorpe Interaction Measure (TIM) and involved videoing a parent-child interaction [32]. Caregivers were asked to look at a picture book with the child in the way that they would at home, stopping either when the child lost interest or became distressed. In the Thorpe Interactive Measure, each picture represents a trial in which the interaction is rated and scores are taken. The primary focus of the rating is the mother’s teaching behaviour (cognitive scaffolding) in showing the picture book to their child. There are six categories of behaviour which are rated: (1) labelling, (2) short elaboration - summarising the content of the picture, (3) long elaboration - including both extension and inference, (4) concept structuring - drawing out concepts such as colour, size, (5) linking - connecting the content of the picture to the child’s own world and experience, (6) child involvement - a range of activities encouraging the active participation of the child. Quality of verbal and non-verbal communication between the mother and child, and the warmth of the relationship is also rated [33]. The static camera recording the caregiver-infant interaction was placed in the upper corner of the room. As a result of this, the caregivers’ and infants’ faces were occasionally not visible, making some judgments difficult. The mean duration of these caregiver-infant interactions was 4.3 (SD = 2.6) minutes with a range from 1.5 to 17.2 minutes. The length of video recordings varied as this was under the control of the mother, or father. The instructions for the TIM were to stop when they felt the child had had enough.

Of these children, 60 were identified after being assessed at 91 months as being likely to have a diagnosis (any oppositional/conduct disorder, any attention deficit hyperactivity disorder, pervasive developmental disorder (autism) or any anxiety or depressive disorder) using the Development and Wellbeing Assessment (DAWBA) [34]. 120 controls were selected with the same sex distribution as the case infants to form a case-control study. For this study we selected the 160 videos where the mother was the lead care giver, 54 of which involved infants that were later diagnosed (based on the DAWBA) with psychopathologies and 106 controls.

### Measures

#### Life event questionnaire

The 44-item Life Event Questionnaire lists a number of events which may have brought changes in the caregivers’ life. They are asked if any of them have occurred since the birth of their child and indicate how much effect it has had on a five point Likert scale ((1) Yes and affected me a lot; 2) Yes, moderately affected; 3) Yes, mildly affected; 4) Yes, but did not affect me and 5) No, did not happen at all). Some of the listed events include: ‘you were in trouble with the law’; ‘you were divorced’; ‘you found that your partner didn’t want your child’; ‘you were very ill’ and ‘your partner lost his job’ (http://www.bristol.ac.uk/alspac/researchers/resources-available/data-details/questionnaires/). The measure has been used in previous studies (i.e., [35]).

#### Social support questionnaire

10-item set of questions which identified the perceived social support of the mother and was adapted by The European Longitudinal Study of Pregnancy and Childhood (ELSPAC) team from work particularly conducted in Greece. The 10-item social support questionnaire includes questions such as: ‘My partner provides the emotional support I need’, ‘I’m worried that my partner might leave me’ and ‘If I was in financial difficulty I know my family would help if they could’. There were four possible responses to each: Exactly feel, often feel, sometimes feel and never feel. The Aggression score was determined by responses on three questions: 1) ‘Does your partner get angry with you?’, ‘Do you have arguments with your partner?’ and ‘Do you get angry with your partner?’. Each had five responses: Almost always, often, sometimes, barely and never (http://www.bristol.ac.uk/alspac/researchers/resources-available/data-details/questionnaires/). The measure has also been used in previous studies (e.g., [36]).

#### Aggression score

The aggression score is derived from three questions which participants have to select one of the following in response: almost always; often; sometimes; barely and never. The three questions are: ‘Does your partner get angry with you?’, ‘Do you have arguments with your partner?’ and ‘Do you get angry with your partner?’ (http://www.bristol.ac.uk/alspac/researchers/resources-available/data-details/questionnaires/). This measure has been used in previous studies (e.g., [37]).

#### Maternal bonding score

Mothers completed a questionnaire regarding maternal bonding at eight months which consisted of two subscales, maternal enjoyment of baby, and maternal confidence subscale. The maternal enjoyment of baby subscale consists of five items for example, ‘I really enjoy my baby’ and ‘it is a great pleasure to watch my baby develop’. The maternal confidence subscale comprises six items including ‘I feel confident with my baby’ and ‘I feel constantly unsure if I’m doing the right thing for my baby’. Participants rate how applicable the statement is to their personal feelings from 1 = never feel to 4 = exact feeling for each of the items. Overall ‘maternal bonding’ score was obtained from combining the two subscale scores with a range of potential scores going from 4–44. The higher the score the greater maternal bonding with the child [38].

#### Mellow Parenting Observation System (MPOS)

The Mellow Parenting Observational System (MPOS; [39]) was used to analysis the videos. Using event recording of positive mother-child interactions, a measure of total positive and total negative interactions is derived. The events recorded included warmth, sensitivity, anticipation and autonomy and the management of distress and control. A number of studies have used the MPOS (e.g., [39]).

The above measures though used in different studies, do not have established psychometric validity or reliability data.

### Procedure

#### Mellow parenting observational system

The quality of relationship between the mother and the infant in the videos were evaluated using the Mellow Parenting Observational System (MPOS) [40]. MPOS coding involves counting of interactions within six categories: Anticipation of Child’s Needs, Autonomy, Cooperation, Responsiveness, Containment of Child’s Distress and Control/Conflict, each of which is scored separately for both positive and negative interactions [39]. For example, in the responsiveness domain, examples of negative parenting include behaviours such as emotional inconsistency, negative affect or criticism. Positive behaviours in this domain include behaviours such as mutual positive affect and maternal affectionate touch. The scores from each category were summed to provide an overall total for both positive and negative interactions. Dividing by the total length of each video gave the rates of positive and negative interactions in counts per minute, which were used as measurements of overall parenting. Observers were blind to case-control status when scoring the videos.

The MPOS was originally developed for families where there were severe relationship problems and around 25% of participating families had a child on the Child Protection Register [41, 42]. Another study also investigated the impact of the mellow parenting programme on later measures of childhood verbal IQ [43]. In the present study, the video quality was relatively poor due to the age of the tapes and the less than optimal camera angles, which may have contributed to the moderate reliability of the MPOS. Given that more reliable measures are expected to be more sensitive (i.e. give higher statistical power), we might expect the use of more modern video equipment to substantially improve the sensitivity of the MPOS.

#### Reliability

The intraclass correlation coefficient (ICC) was used to assess inter-rater reliability for the rate of total positive interactions. Measures with ICC > 0.5 were deemed reliable [44]. Given the non-normal distribution of the rate of negative interactions, a non-parametric measure of reliability, Kendall’s τ, was used to investigate agreement between the different raters. Kendall’s τ determines the concordance among the ranks as opposed to the measures themselves. Measures with τ > 0.6 were defined as reliable.

#### Variable selection

From the data set available from ALSPAC, a reduced group of twenty predictor variables were selected, by investigator consensus, on the basis of previous literature and face validity. These included parental and infant characteristics, indicators of parental socio-economic status (SES) and maternal pre- and post-natal emotional state (Table 1). The Bonding Scale was delivered at eight weeks and consisted of 11 questions to examine how the mother felt about looking after the baby. It gave four options from ‘This is exactly how I feel’ to ‘I never feel this way’. Anxiety was measured in pregnancy and postnatally using the free floating anxiety subscale of the Crown-Crisp Experiential Index (CCEI) [45]. Depression was measured at the same time points as anxiety using the 10-item Edinburgh Postnatal Depression Scale (EPDS) [46]. The Mini International Neuropsychiatric Interview (MINI) [47] suggests that a cut-off score of 12 is optimal to detect the presence of depression and this cut-off score was adopted in the present study.

**Table 1:** **Univariate associations of predictors with the rate of positive and negative interaction scores**

		Summary statistics for predictor*	Associations with rate of negative interactions	Associations with rate of positive interactions
Child Gender	Female	49 (30.6%)	-	-
	Male	111 (69.4%)	1.71 (0.81, 3.62), p = 0.160	0.89 (0.74, 1.06), p = 0.202
Mother Age at birth (for 1 year increase)		29.5 (4.5)	**0.90 (0.83, 0.97), p = 0.004**	**1.02 (1.00, 1.04), p = 0.033**
Parity (per unit increase)		0.7 (0.8)	0.87 (0.56, 1.36), p = 0.550	0.97 (0.88, 1.08), p = 0.584
Maternal depression at 32-40 weeks (per unit increase)		6.9 (5.0)	1.01 (0.94, 1.08), p = 0.812	1.01 (1.00, 1.03), p = 0.118
Postnatal depression at 8 months (per unit increase)		5.6 (5.0)	1.03 (0.97, 1.10), p = 0.354	1.01 (0.99, 1.02), p = 0.478
Maternal anxiety at 32-40 weeks (per unit increase)		4.7 (3.4)	1.03 (0.93, 1.14), p = 0.630	1.02 (0.99, 1.04), p = 0.153
Postnatal anxiety at 8 months (per unit increase)		3.8 (3.9)	1.00 (0.92, 1.10), p = 0.934	1.01 (0.99, 1.04), p = 0.172
Infant breast fed	No	24 (15.1%)	-	-
	Yes	135 (84.9%)	1.26 (0.47, 3.36), p = 0.649	1.19 (0.94, 1.51), p = 0.150
Marital status	Never married	22 (13.8%)	-	-
	1^st^ marriage	123 (77.4%)	1.09 (0.40, 2.97), p = 0.873	1.27 (1.00, 1.63), p = 0.054
	2^nd^/3^rd^ marriage	9 (5.7%)	1.03 (0.18, 5.82), p = 0.970	1.25 (0.82, 1.90), p = 0.292
	Divorced	5 (3.1%)	0.66 (0.07, 6.16), p = 0.718	1.34 (0.80, 2.24), p = 0.264
Father in household	No	14 (9.2%)	-	-
	Yes	139 (90.8%)	0.50 (0.15, 1.63), p = 0.251	1.20 (0.89, 1.62), p = 0.225
Maternal education levels	Vocational/CSE/GCSE	89 (56.0%)	-	-
	A level/Degree	70 (44.0%)	1.02 (0.51, 2.04), p = 0.958	**1.32 (1.12, 1.55), p = 0.001**
Anyone with chronic illness in household	No	133 (88.7%)	-	-
	Yes	17 (11.3%)	1.11 (0.36, 3.42), p = 0.861	0.89 (0.68, 1.16), p = 0.389
Smoked during first trimester	No	128 (81.0%)	-	-
	Yes	30 (19.0%)	0.64 (0.26, 1.58), p = 0.331	0.91 (0.73, 1.13), p = 0.384
Alcohol during first trimester (glasses of alcohol per week)	< 1	129 (81.6%)	-	-
	≥ 1	29 (18.4%)	1.04 (0.43, 2.52), p = 0.929	1.04 (0.84, 1.29), p = 0.737
Partner physically hurt mother at 18 weeks gestation	No	143 (93.5%)	-	-
	Yes	10 (6.5%)	1.29 (0.32, 5.17), p = 0.718	1.03 (0.72, 1.46), p = 0.880
Partner physically hurt mother postnatally	No	152 (95.0%)	-	-
	Yes	8 (5.0%)	0.34 (0.06, 1.97), p = 0.230	1.01 (0.69, 1.47), p = 0.962
Social support score (per unit increase)		20.1 (4.8)	0.94 (0.87, 1.01), p = 0.072	1.01 (0.99, 1.03), p = 0.335
Life event score 18-23 weeks (per unit increase)		8.6 (6.5)	1.02 (0.97, 1.08), p = 0.417	1.00 (0.98, 1.01), p = 0.716
Maternal bonding score (per unit increase)		28.0 (4.0)	0.98 (0.91, 1.07), p = 0.723	**0.98 (0.96, 1.00), p = 0.024**
Aggression score (per unit increase)		10.2 (1.8)	0.96 (0.78, 1.17), p = 0.655	1.03 (0.98, 1.08), p = 0.300

*Mean (SD) presented for continuous variables and N (%) for categorical.

- indicates reference category in regression analysis.

(Effect estimates are the relative change in interaction scores for a specified increase in continuous predictor variables or compared to the stated reference group for categorical predictors).

Statistically significant associations (p<0.05) are highlighted in bold text.

### Statistical methods

#### Regression analysis

We used negative binomial regression models to examine the association between the predictors and the rate of positive and negative interactions. The counts of interactions were modelled as the outcome variables with the log video duration as an offset term. Backward stepwise selection was used; starting with all potential predictors in the model, at each step one of the predictors was removed, based on the greatest improvement in the Akaike Information Criterion (AIC), ensuring that the effect estimates were significant at the 10% level, until a model was reached where no predictors could be removed without increasing the AIC.

#### Caseness

By the nature of the design, this sub-sample had an inflated rate of cases as compared to the original population, with one third known to develop psychopathology at around age 7, compared to 4.8% of the overall number who attended the ‘Children in Focus’ clinics. To assess if this had an effect on variable selection to the model we included infant diagnostic outcome – either case or control – as a predictor. We compared the variables included in the final model by either using model selection that did not consider diagnostic outcome, or by retaining diagnostic outcome in the model throughout variable selection. We also examined interactions between diagnostic outcome and the final model variables.

All statistical analysis was performed using R statistical package v2.15 [48].

## Results

Measurements of the rate of positive interactions were moderately reliable with an inter-class correlation of 53%. Measurements of the rate of negative interactions had a correlation of 0.60 using Kendall’s τ.

Within the Mellow Parenting Observational System, observations of positive and negative interactions are counted within six domains. It was notable that some domains had very low levels (fewer than 10% of videos) with non-zero counts, and the inter-rater reliability for many individual domains was poor. This is in contrast to the reasonable reliability found for the total positive and negative scores, suggesting that whilst raters were able to detect positive and negative interactions, they were less able to differentiate between different dimensions of interaction with these videos. We carried out an exploratory factor analysis of the separate positive and negative interaction domains, but found no evidence that there was an underlying factor structure that adequately explained the data. We have therefore presented the results of analyses based on the total positive and negative interaction scores alone.

Table 2 summarises the total counts of positive and negative interactions in the 160 videos analysed, as well as the corresponding rates of interactions in counts per minute, and the durations of the videos themselves. Videos lasted between one and 8.5 minutes. While positive interactions were observed in all videos, occurring at a mean rate of 6.2 for each minute of video, there were few negative interactions recorded; in 103 (64%) of the videos, no negative interactions were identified.

**Table 2:** **Mean (SD) of interaction scores on 160 subjects**

Total positive interaction counts	20.65 (12.30)
Total positive interaction rate (counts/min)	6.22 (3.30)
Total negative interaction counts	1.13 (2.22)
Total negative interaction rate (counts/min)	0.37 (0.77)
Video duration (seconds)	210.91 (86.19)

Table 1 summarises the potential predictors of positive and negative interactions, and shows the univariate associations between each variable and the rate of positive and negative interactions, expressed as relative effect estimates. Older mothers had more positive and fewer negative interactions with their infant and higher levels of maternal education and maternal bonding scores were associated with an increased rate of positive interactions.

Backward stepwise regression analysis identified four variables that independently predicted the rate of negative interactions (Table 3). Fewer negative interactions were observed with older mothers, mothers who perceived that they received more social support during pregnancy (encompassing perceived emotional and financial support from a partner, friends, family, neighbours, other pregnant women and the state), mothers who smoked during the first trimester and mothers with female infants.

**Table 3:** **Relative effects of each predictor variable on the rate of interactions between the mother and infant; results of backwards stepwise regression**

	Effect estimate (95% CI)	p-value
**Negative interactions (N = 153)**		
Mother age at birth (years)	**0.90 (0.83, 0.97)**	**0.007**
Male child gender	**2.19 (1.03, 4.66)**	**0.041**
Social support score	**0.92 (0.85, 0.98)**	**0.013**
Smoked during first trimester	**0.30 (0.11, 0.78)**	**0.014**
**Positive interactions (N = 154)**		
Mother age at birth (years)	1.02 (1.00, 1.04)	0.061
Maternal education level: A level or Degree (vs. Vocational/CSE/GCSE)	**1.29 (1.09, 1.53)**	**0.003**
Maternal anxiety at 32-40 weeks	1.02 (1.00, 1.05)	0.051

(Effect estimates are the relative change in interaction scores for a specified increase in continuous predictor variables or compared to the stated reference group for categorical predictors).

Statistically significant associations (p<0.05) are highlighted in bold text.

Three variables were found to be independent predictors of positive interactions. Higher rates of positive interaction were observed with older mothers, mothers with a higher level of education and mothers who experienced anxiety during the third trimester. Maternal age and anxiety did not quite reach conventional levels of statistical significance, but excluding either predictor led to an increase in the model AIC, indicating a poorer model fit. In exploratory subgroup analysis we examined whether the association between anxiety and positive interactions remained for mothers who also exhibited depressive symptoms. We categorised the depression score into two groups; from 0 to 12 and greater than 12, indicating potential depressive symptoms. Increased positive interactions were only associated with increased anxiety in mothers who did not show depressive symptoms (depression – anxiety interaction p-value =0.052).

Diagnostic outcome at 91 months was not associated with the rate of negative interactions and the addition to the model of an indicator for caseness did not alter the coefficients of the other variables. There were no interactions between diagnostic outcome and the other predictors. Diagnostic outcome was however associated with lower rates of positive interactions. Moreover, we found an interaction between becoming a case and the association between maternal anxiety and positive interactions (interaction p-value, 0. 022); for those infants who went on to develop psychopathology, there was no evidence of an association (relative effect estimate: 1.00 (0.98, 1.02); p = 0.932), but amongst the control infants, the rate of positive interactions was increased in mothers who had greater pre-natal anxiety (1.06 (1.02, 1.09); p = 0.001). Given that the controls are relatively under-represented in our sample, this suggests that the association between maternal anxiety and positive interactions is underestimated in the model shown in Table 3. No other interactions were found, and the addition of the diagnostic outcome to the model did not affect the coefficient estimates for the other predictors. The same variables were identified as predictors when diagnostic outcome was included and when excluded from the model selection process. These results indicate that the modelling results are robust to the sample construct.

## Discussion

### Summary of main findings

Using data from a nested case-control study within a large community-based cohort of infants, various pre- and post-natal variables were entered into a regression analysis to ascertain the predictors of parenting at one year according to the Mellow Parenting Observational System. A positive association between prenatal ‘free floating’ anxiety levels (anxiety not confined to specific situations or issues) and future positive parenting was found, which has not previously been reported in the literature. From this it could be postulated that increased anxiety during late pregnancy has an adaptive rather than detrimental effect as has previously been reported [2]. Maternal age at delivery was found to be a significant predictor in both models, with younger mothers exhibiting higher rates of negative interaction and lower rates of positive interactions. This adds clarity to previous literature, where there have been conflicting findings relating to younger maternal age and parenting styles [2, 15–17].

An interesting and novel finding of our study was that male children were more likely to be negatively parented, an observation not previously reported in a community sample. Being a male is likely associated with being a case (ADHD, OCD and autism are more common in boys), and if negative parenting is associated with being a case, then by extension, being a male will be associated with negative parenting. However, the relationship with negative parenting held even when controls were analysed separately. Although this may not be useful as a predictor in terms of public health policy, it could have implications due to the known link between male gender and likelihood of developing conduct disorder [14]; it could be that parenting behaviours mediate this increased risk.

### Limitations and strengths of the study

The main limitations of the study relate to the quality of the videos and the nature of the sample used. The videos were rather unhelpful in terms of the visibility of some interpersonal behaviours, and the quality of the tapes was relatively poor. It is also noted that counts of negative parenting were low throughout the sample, which may be due to the artificial setting. The low number of negative interactions in the videos is also a potential limitation due to the limited power achievable as a consequence. Regarding specifically the finding of a positive association between prenatal anxiety levels and future positive parenting, these findings should be treated with caution given that they were only of marginal statistical significance.

As the dataset originates from a nested case-control study the inflated number of cases could be seen to confound results. Steps taken to account for this were the inclusion of caseness as a predictor variable in regression analyses and the testing for interactions with other explanatory variables. We found evidence that the observed association between maternal anxiety during late pregnancy and the rate of positive interactions is underestimated in the sample as a whole, and we cannot exclude the possibility that other factors associated with parenting behaviour may have been obscured by the study design, or simply missed due to the moderate sample size. Nevertheless, we were able to identify a number of factors, mostly measured during pregnancy, that were associated with both positive and negative interactions between mothers and their one-year-old infants, despite the nature of the study sample used.

Lastly, the findings have to be accepted cautiously and have to be replicated, as the measures used do not have established psychometric validity and reliability data.

### Wider findings and implications for clinical practice

In relation to predictors of positive parenting, the final model included variables which both support and add to the previous evidence base. The findings relating to maternal age at delivery add clarity to debate within the literature, and support the claim that younger mothers may need more support and are a group which may potentially gain particular benefit from parenting interventions. Higher levels of maternal education were shown to be associated with more positive parenting behaviours, findings which support the existing evidence base [20]. The positive association between pre-natal anxiety and positive parenting is a new finding, and, if confirmed in other studies, should be taken into consideration when advising and potentially reassuring women who present as being particularly anxious during their pregnancy.

In the final regression model for negative parenting, the negative association seen with maternal age at delivery reinforces the importance of supporting young mothers, who are both less likely to positively parent and more likely to negatively parent. The negative association between social support score and negative parenting behaviours highlights the need to establish a woman’s perceived support during their pregnancy, and the importance of identifying isolated mothers who may be more likely to negatively parent their child.

The association between male gender and negative parenting is striking. The scale of the association, with male infants having more than twice the rate of negative interactions, makes it a significant finding in its own right. It may be this is in some way related to the fact that all primary caregivers featured in the videos were mothers, but given the lack of existing research around this topic any such inferences at this stage would be speculative. If our findings are confirmed, it may be possible to make a case for offering more parenting support to mothers of boys.

In this sample, an association was found between non-smoking and negative parenting, which conflicts with much of the literature on this topic [29]. In contrast to our results, some studies using data from ALSPAC have found that maternal self-reported smoking contributed to the prediction of poor child development [49] and it has been suggested that smoking during pregnancy should be considered in identifying women and their offspring likely to benefit from parenting support interventions [50]. However, the number of smokers within the sample used for this particular study was small (n = 30, 19% of total sample size) and when taken into consideration alongside the frequency of zero counts for negative parenting (n = 103, 64% of total sample size) and changed attitudes and policy towards smoking during pregnancy since 1991 [51], given the much higher public awareness of the dangers of smoking in pregnancy now, it is possible that this finding is an artifact, or representative of this particular population or time period.

Many variables included in the initial regression analysis which were found to be non-significant in this population are factors which have previously been well established in the literature: specifically maternal depression [2, 6, 19, 24, 25], marital status [17] and social class [2, 15, 22]. It could be argued that within this population other variables included were acting as proxies for these expected variables, or indeed that in previous studies some of the association seen was in fact masking an association with another variable which was simply not being measured as part of the study design.

## Conclusions

From this study, the factors which appear most important in identifying those families most at risk of negatively parenting their child are decreased age of the mother at delivery and lack of perceived social support during pregnancy. Mothers who report achieving lower levels of education appear to be less likely to parent positively. Mothers who experience anxiety in late pregnancy may actually engage in more positive interactions with their infants, and male children appear to be more likely to be negatively parented, at least by their mother.
